# Long-term use of a shark breeding ground: Three decades of mating site fidelity in the nurse shark, *Ginglymostoma cirratum*

**DOI:** 10.1371/journal.pone.0275323

**Published:** 2022-10-17

**Authors:** Harold L. Pratt, Theo C. Pratt, Ryan J. Knotek, Jeffrey C. Carrier, Nicholas M. Whitney

**Affiliations:** 1 Anderson Cabot Center for Ocean Life, New England Aquarium, Boston, Massachusetts, United States of America; 2 Elasmobranch Field Research Association, South Thomaston, Maine, United States of America; 3 Department of Biology, Albion College, Albion, Michigan, United States of America; Institut de Recherche pour le Developpement, FRANCE

## Abstract

Understanding shark mating dynamics and mating site use may be vital to species management. The Dry Tortugas courtship and mating ground (DTCMG) has been known as a mating site for nurse sharks, *Ginglymostoma cirratum*, since 1895. In a 30-yr (1992–2021) study we have documented long-term site fidelity to this area with data from 137 adult sharks (89 female, 48 male) tagged with PIT, fin, and acoustic tags. Of 118 sharks tagged from 1993 to 2014, at least 80 (68%) returned to the DTCMG in subsequent years during the June-July mating season. Known individuals returned in up to 16 different mating seasons and over periods of up 28 years, indicating that life span extends well into the forties for this species. Of all returning sharks, 59% (N = 47) have been monitored for over 10 years and 13% (N = 10) have been monitored for over 20 years. Males arrived annually in May and June and departed in July, whereas females arrived biennially or triennially in June, with a secondary peak in site use in September and August, likely associated with thermoregulation during gestation. During the mating season, males made more frequent visits of shorter duration (median = 34 visits for 1 h per visit) to the DTCMG, whereas females made fewer visits but remained on site for longer periods (median = 12.5 visits for 4.4 h per visit). Females typically mated biennially but showed a triennial cycle in 32% of cases, with many females switching cycles at least once. This pattern would reduce the potential reproductive lifetime output of a female by 11% compared to what would be projected from a strict biennial cycle. The long-term mating site fidelity of this shark population reveals the importance of identifying and protecting mating sites for this and other elasmobranch species.

## Introduction

The life histories of sharks and rays typically include restrictive features such as slow growth and low fecundity that make them particularly vulnerable to fishing pressure [1–3], and over a third of chondrichthyans are now threatened with extinction [4]. Understanding the influence of reproductive behavior on fish movement and habitat selection has been recognized as crucial for proper management of teleost species [e.g., 5–8] but is far less understood in elasmobranchs. Although the repeated use of nursery [9–11] and gestation areas [12–15] has been documented for several shark species, relatively few consistent breeding grounds have been identified [16, 17].

For most shark species, breeding grounds are unknown or inferred from adult movements [18–20], occasional observations of mating at annual aggregation sites [21], or fresh mating wounds observed at specific times and locations [22, 23, reviewed by 24]. For pelagic species, oceanic fronts may be more important for breeding synchronization than a specific geographic location [25], whereas consistent breeding and oviposition sites are well known for some smaller benthic species [26–28]. A recent, multi-year study of Port Jackson sharks (*Heterodontus portusjacksoni*) showed that most tagged adults returned to the same bay, and often the same site within that bay, to breed every year over the 3 to 4 year monitoring period [29], emphasizing the importance of specific breeding grounds for that species.

Despite our poor understanding of the location and usage of shark breeding grounds, any area that produces a species aggregation is likely to have disproportionate management implications [16] because it either represents essential habitat [e.g., 14, 30] or increases the risk of fisheries exploitation [16, 31]. For this reason, identifying additional shark breeding sites and understanding the usage of known sites are important fields of study [21, 32].

The Dry Tortugas courtship and mating ground (DTCMG) for nurse sharks (*Ginglymostoma cirratum*) is likely the most well-documented shark breeding ground in the world. Nutting [33] first reported mating nurse sharks in the Dry Tortugas in 1895, and Gudger [34] later described mating here and dissected an ovigerous female in 1912. Klimley [35] confirmed mating at the Dry Tortugas and documented courtship and mating of nurse sharks at the Miami Seaquarium. Over the last 30 years, work by the authors in the DTCMG has described the dynamics of nurse shark courtship and mating behavior [24, 36–38], assessed their reproductive status using imaging techniques [39], and characterized the habitat of the breeding ground [40]. Carrier and Pratt [41] documented the need and successful implementation of management and protection for the DTCMG, making it the only marine protected area designated specifically for a shark breeding ground. More recent work has used modern biologging technology to quantify nurse shark mating behavior [42] and acoustic telemetry to document their migrations [43].

The nurse shark is a large, coastal species reaching 3 m in length [44] found in subtropical/tropical waters on both sides of the Atlantic and feeds on a broad range of fish and invertebrate taxa [45]. Although they have a low metabolic rate [46] and are thought to be relatively restricted in their movements [17, 47–49], recent work has shown that these sharks are capable of repeated, long-distance migrations of several hundred kilometers [43] to and from the DTCMG.

Direct observational or tagging research on this nurse shark population in the DTCMG has taken place every year from 1992 to 2021. In June and July, female nurse sharks aggregate in clear, shallow water where they are approached by males for courtship and mating, making this a rich site for behavioral observation and tagging studies [24]. The goal of the current study was to examine our collective 30 years of resighting and tagging data to present a detailed picture of how nurse sharks utilize the DTCMG. Specifically, we examined the differential usage of the breeding ground by male and female nurse sharks over multiple time scales, including 1) over periods of multiple years, 2) within the calendar year, and 3) within the mating season.

## Methods

### Study site

**Fig 1:**
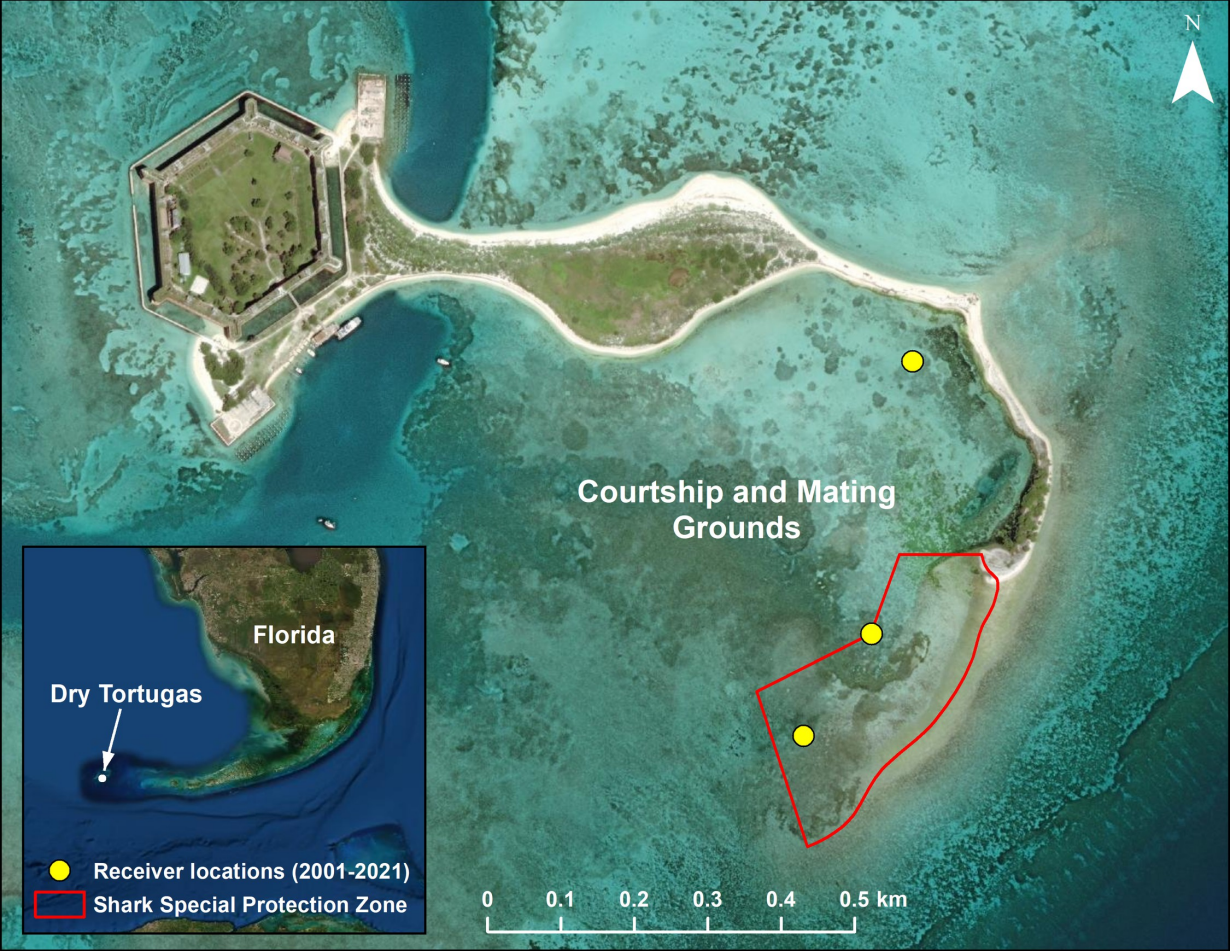
The Dry Tortugas courtship and mating ground with year-round acoustic receiver locations from 2001 to 2021. Receivers shown as yellow circles, the general area used by nurse sharks during the mating season (denoted with text), and the Shark Special Protection Zone (red outline) that has been closed to human activity during the mating season since 1998. Inset shows the location of Dry Tortugas relative to the Florida peninsula. The basemap (world imagery) image is the intellectual property of Esri and is used herein under license. Copyright © 2020 Esri and its licensors. All rights reserved. Basemap source: Esri, Maxar, Earthstar Geographics, and the GIS User Community.

The DTCMG study site covers most of a ~20-hectare lagoon in the Dry Tortugas bracketed by Bush Key, Long Key, and the Long Key intertidal bar (Fig 1). Most nurse shark courtship and mating activity has been observed in a small and shallow (5 hectare; 0 to 1.8 m deep) section of the DTCMG consisting of a sand and seagrass flat (described in detail by [40]). This smaller area, known as the Shark Special Protection Zone, has been seasonally closed to boat traffic during the shark reproductive season for over 20 years by the National Park Service [41]. Adult female nurse sharks aggregate in these shallows each year, often shoaling in less than 1 m of water. Mating typically occurs in these shallows or nearby in slightly deeper (2 to 3 m) water. This annual aggregation in shallow, clear water makes the site ideal for tagging sharks and monitoring their behavior for prolonged periods [24].

### Capture and measurement

**Fig 2:**
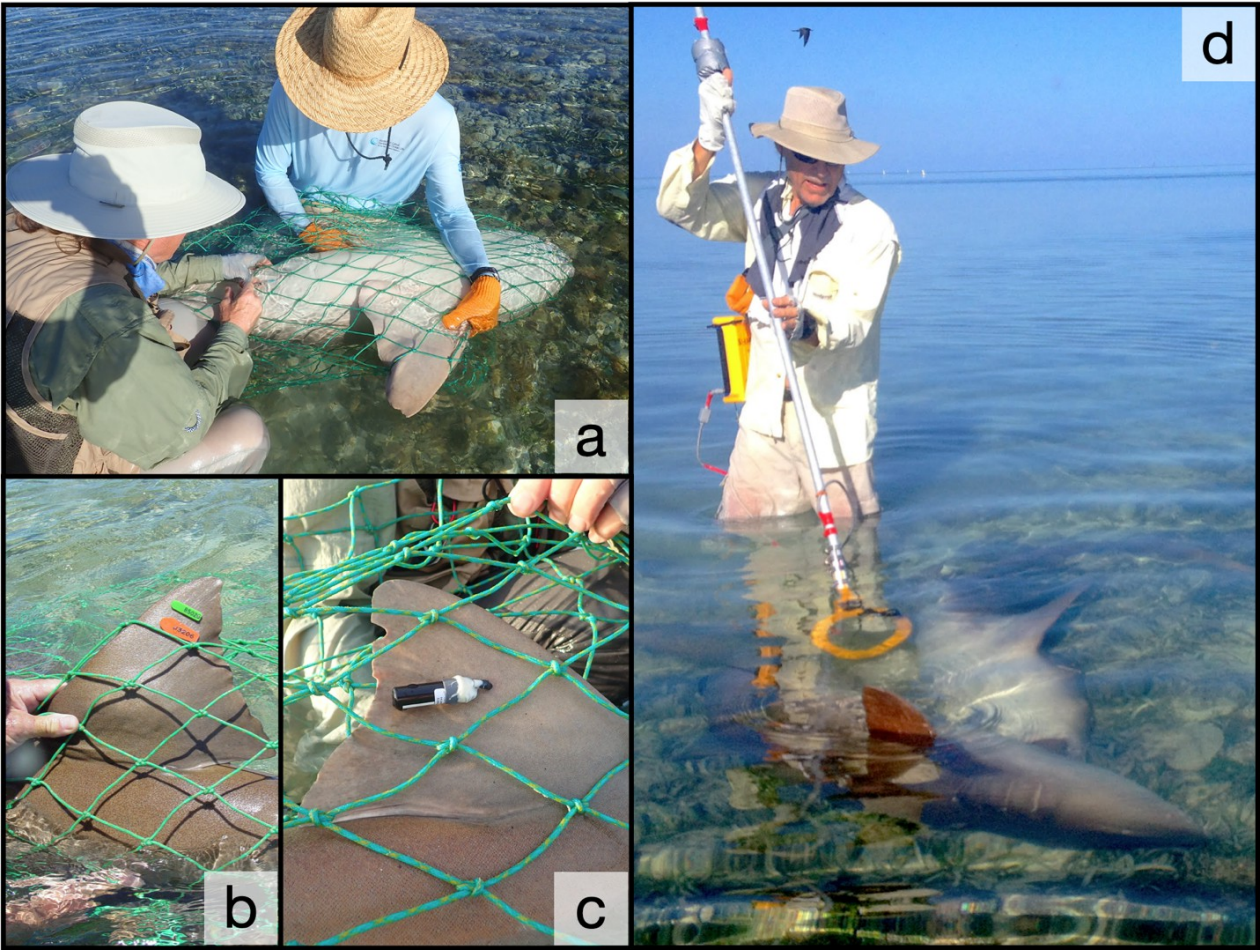
Capture, tagging, and identification of nurse sharks in the Dry Tortugas courtship and mating ground. Nurse sharks are captured in custom hoop nets and a) held in the shallows inside the capture nets where researchers can attach b) fin-mounted rototags, c) acoustic transmitters, and a) hold animals in tonic immobility for surgical implantation of transmitters. Sharks mate in clear, shallow waters where they can d) be approached and scanned with a submersible PIT tag reader (yellow ring) to obtain shark ID’s. Image (d) was printed with permission from R. Rose, copyright 2022.

Sharks were captured by hand and tagged using techniques described in Pratt and Carrier [38]. Briefly, sharks were approached on foot while resting, courting, or mating in the shallows (< 1 m) and captured in large, custom-made dip nets (80 x 320 cm) that also served as holding bags for the tagging process (Fig 2A). Nurse sharks are buccal breathers capable of pumping water over their gills while resting in place, so they are typically calm while being restrained in their natural habitat during measurement and tagging. Sharks were measured in girth behind the pectoral fins forward of the first dorsal, and in total length (TL) [50] along the midline to the tip of the tail while in the net. This study was conducted under research permit provided by the National Park Service at Dry Tortugas and Everglades (#DRTO-2020-SCI 0005), and all sampling followed applicable international, national, and/or institutional guidelines for the care and use of animals. The research was carried out under Institutional Animal Care and Use Committee (IACUC) protocol #2019–05 at New England Aquarium and protocol #FL_DRTO_Whitney_NurseShark_2021.A2 at the National Park Service.

### Identification and tagging

Photograph and video records for the identification of unique fins started in 1992. From 1993 to 1999, some sharks were dart tagged with a modified spear gun. Every shark captured since 1994 has received a passive integrated transponder (PIT; Biomark, Inc., Idaho, USA; FDX RFID 12 × 2 mm biocompatible glass 125 kHz) under the epidermis on the right-hand side of the first dorsal fin base and one or more nylon rototags (Dalton Tags, Nottinghamshire, UK) [51] on either the first or second dorsal fin for females and males, respectively (Fig 2B). In 2001, we began tagging nurse sharks with acoustic transmitters using methods described in Pratt et al. [43]. Acoustic transmitters (Innovasea Systems, Inc., Halifax, Nova Scotia; V16-6H transmitters, 16 x 95 mm, 36 g in air) had a nominal transmission delay of 240 s with a maximum life of 3650 d. Transmitters were attached externally to the dorsal fins of females (Fig 2C), and most males were tagged internally via surgical implant (Fig 2A) through a small (25 to 30 mm) abdominal incision on the ventral side of the shark along the midline, anterior to the pelvic fins and siphon sac. The tag was placed inside the peritoneum, and the incision closed with 3 to 4 interrupted sutures (Ethicon Inc., New Jersey, USA; 2–0 PDS II [43]). Surgical implantation was avoided for females to reduce the risk of impacting gestation.

Nurse shark identification in subsequent years involved one or more methods for confirming the presence and identity of sharks. Captured sharks were easily identified based on external tags, fin markings, or PIT tag IDs. Free-swimming sharks were identified via visual confirmation of external tags or fin markings, reading of PIT tag ID with a Destron Fearing (Texas, USA) FS2001 reader with racket antenna (Fig 2D), or by detection via acoustic receivers.

### Acoustic telemetry

Three omni-directional acoustic receivers (Innovasea Systems, Inc.; VR2 and VR2W) were deployed in the DTCMG (Fig 1) and downloaded at least once a year from 2001 through 2021. The DTCMG is a shallow (0–2 m) area enclosed by land on three sides, and the acoustic detection range is known to vary widely in these conditions [52]. The receivers inside the DTCMG were set up for detection redundancy to account for this varied environment and were considered one station for analytical purposes. Acoustically tagged sharks traveling outside the DTCMG array were frequently detected by two larger arrays consisting of 74 total receivers stationed throughout the larger Dry Tortugas by collaborators focusing on fish [53] and sea turtle [54] movements from 2008 to 2013. Fine-scale spatial use of these additional sites is beyond the scope of this study. However, we pooled these detections to confirm whether animals remained in the greater Dry Tortugas with functioning tags when not detected within the DTCMG.

### Data analysis

#### Long-term annual use of the DTCMG

Nurse shark usage of the DTCMG during the mating season was evaluated over multiple years by considering all identification methods described above (i.e., visual confirmation of external tags and fin markings, reading of PIT tag IDs, and acoustic detections) to confirm presence within a mating season. Only sharks tagged through 2014 were included in our long-term evaluation to focus on individuals that have been at large long enough to establish a long-term return pattern. We counted the number of mating seasons each shark was detected and quantified time-at-large as the elapsed time (in years) between each shark’s first and most recent identification. The number of mating seasons and time-at-large between sexes was compared using the Mann-Whitney U test (“stats” package in R [55]). This and all other analyses were performed using R 4.2.1 [55], and statistical significance was accepted at a p-value < 0.05.

The periodicity of nurse sharks returning to the DTCMG in the mating season was examined by calculating the time (in years) between returns for each individual. An Anderson-Darling test (“twosamples” package in R [56]) was used to compare the density distributions of time between returns for males and females and evaluate periodicity between the sexes. Generalized linear mixed effect models (GLMM) with a binomial distribution and logit link function were used to examine the proportion of return events across periods (i.e., the time between returns). Nurse shark IDs were treated as the random effect to account for variability and repeated observations of an individual, and models were run separately for each sex. GLMMs were implemented in a Bayesian setting via the *blgmer* function of the “blme” package in R [57] to account for complete and quasi-complete separation within our data (e.g., periods containing no return events by any individuals). This approach sets weakly informed priors on the fixed effect coefficients to facilitate proper estimation. Wald chi-square tests (“car” package in R [58]) were used to test the overall effect of the period from the fitted model, and post-hoc multiple comparisons were performed using Tukey’s p-value adjustment (“emmeans” package in R [59]) to identify which period(s) displayed similar nurse shark return proportions.

#### Within-year use of the courtship and mating ground

Only acoustic detection data were used to characterize shark usage of the DTCMG throughout the calendar year since all other observation methods were restricted to sampling during the mating season. All detection data were binned by day and plotted by the individual shark for males and females separately. We evaluated the seasonality of nurse sharks arriving and departing from the site using strings of acoustic detections consisting of two or more observations, wherein the first detection (an arrival event) followed a ≥ 30-day absence and the final detection (a departure event) preceded a ≥ 30-day absence. Strings of arrivals and departures from the time of tagging until the following April were excluded to remove any bias associated with tagging. The April threshold was used since mating season arrivals typically begin in May. Anderson-Darling tests were used to compare density distributions of arrivals or departures between sexes to determine whether similar patterns were displayed. GLMMs with a binomial distribution and logit link function were used to evaluate the proportion of arrivals or departures by month throughout the year. Nurse shark ID and year were treated as random effects to account for repeated observations and variability between individuals and any inter-annual variability. Models were run separately for each sex and performed in the Bayesian setting to account for separation issues within the proportional data across several months. We also tested the overall effect of the month as a factor and performed post-hoc multiple comparisons using the methods previously described for GLMMs.

#### Within-season use of the courtship and mating ground

Usage of the DTCMG within the mating season was characterized by using all acoustic detection data except for the initial tagging year to avoid any sampling bias. Individual visits to the site were characterized as a single detection or series of detections preceded and followed by at least 60 minutes without any detections. These visits represent fine-scale site usage within the timeframe between arrival and departure events. Site usage information was compiled across all sharks and years and compared between sexes according to four metrics: the (1) number of visits, (2) visit duration, (3) cumulative time spent on site, and (4) time from arrival to departure (defined in the previous section). We examined visit duration based on individuals’ mean visit durations within each mating season. This approach equally weighted mating seasons across individuals so that our comparison was robust to differences across seasons and not biased by any individual visits made by a shark. Cumulative time spent on site was calculated by summing visit durations within each season for individuals. Metrics were compared between sexes using GLMMs with either a Poisson distribution and log link function (i.e., for the number of visits only) or Gaussian distribution and identity link function. Nurse shark ID was treated as a random effect in all models to account for repeated observations and individual variability.

Daily site usage in the mating season was also visually examined using the mean and standard deviation of hourly detection proportions across each mating season, excluding detections from initial tagging years. We assessed the potential influence of seawater temperature on daily site usage by visually comparing hourly detection proportions to the mean and standard deviation of hourly on-site temperatures throughout the mating season.

## Results

We tagged 137 adult nurse sharks (89 female, 48 male) with PIT and fin tags (usually rototags) on the DTCMG from 1993 to 2021 (Table 1). A subset of 76 sharks (43 female, 33 male) was tagged with acoustic transmitters from 2001 to 2014. Tagging effort was not evenly distributed across years, and annual return patterns for sharks tagged in recent years are not yet known, so sample sizes varied between analyses and are reported in each case. From all tagging and resighting methods combined, we have detected an average of 26 ± 4 sharks per year on the DTCMG over the last ten years (2012–2021). We collected 211 unique length-year measurements (many sharks were measured multiple times but only once per year) and the mean ± SD, and range of TL values were 250 ± 10, 220–275 cm TL (N = 142 measurements) for females and 242 ± 10, 219–266 cm TL (N = 69) for males. Females also showed consistently larger girth measurements (105 ± 5, 91–119 cm; N = 100) than males (87 ± 5, 76–96 cm; N = 53).

**Table 1:** Nurse shark tagging effort in the Dry Tortugas courtship and mating ground from 1993 to 2021. For each sex, the total number of tags (PIT, Roto tag, and acoustic transmitter) deployed each year, and the cumulative number of deployed tags (in italics) are provided. In addition, Roto and acoustic transmitter tags that were re-deployments (i.e., on animals that had previously been tagged) are provided inside parentheses for the respective year. These re-deployments were not included in the cumulative number of deployed tags. Dashes indicate instances where no sharks were tagged with a tag type deployed in a given year, whereas grey cells indicate years where certain tag types were not yet deployed in this study.

	PIT			Roto			Acoustic		
Year	Female	Male	Total	Female	Male	Total	Female	Male	Total
1993^a^									
1994	-	1	1	1	1	2			
1995	-	1	2	1	2	5			
1996	1	-	3	2	-	7			
1997	6	1	10	5 (1)	-	11			
1998	-	-	10	1 (1)	-	11			
1999	-	-	10	-	-	11			
2000	13	1	24	11	2 (1)	23			
2001^b^	4	6	34	4	4	31	2	6	8
2002	5	4	43	3	4	38	5	4	17
2003	9	4	56	8 (1)	4	49	2	4 (1)	22
2004 ^c^	7	2	65	9 (2)	3 (1)	58	6	5 (3)	30
2005	5	5	75	7	5	70	9 (2)	8 (3)	42
2006	4	1	80	4	2 (1)	75	4	2	48
2007	5	1	86	6	1	82	5 (2)	1	52
2008	2	3	91	4 (2)	4	88	6 (3)	4 (2)	57
2009	7	2	100	8 (2)	2	96	7 (1)	2	65
2010	3	3	106	3	4 (1)	102	4 (1)	4 (1)	71
2011	2	1	109	3 (1)	1	105	1	-	72
2012	4	-	113	5 (1)	-	109	-	-	72
2013	-	3	116	1 (1)	3	112	-	4 (1)	75
2014	-	-	116	1 (1)	-	112	4 (3)	-	76
2015	-	-	116	-	-	112	-	-	76
2016	-	-	116	1 (1)	-	112	-	-	76
2017	-	-	116	-	-	112	-	-	76
2018	2	1	119	3 (1)	1	115	-	-	76
2019	5	1	125	6 (1)	1	121	5 (2)	2 (1)	80
2020	4	2	131	5 (1)	3 (1)	127	5 (1)	3 (1)	86
2021	1	5	137	1	6 (1)	133	5 (4)	4 (1)	90
**Total**	89	48	137	84	47	133	51	39	90

^a^Two sharks were identified in 1993 by dart tagging or photographing unique fin markings on free-swimming animals.

^b^No sharks were tagged with acoustic transmitters prior to 2001.

^c^Three females were tagged in October 2004 outside of the mating season and did not return.

### Long-term annual use of the courtship and mating ground

**Fig 3:**
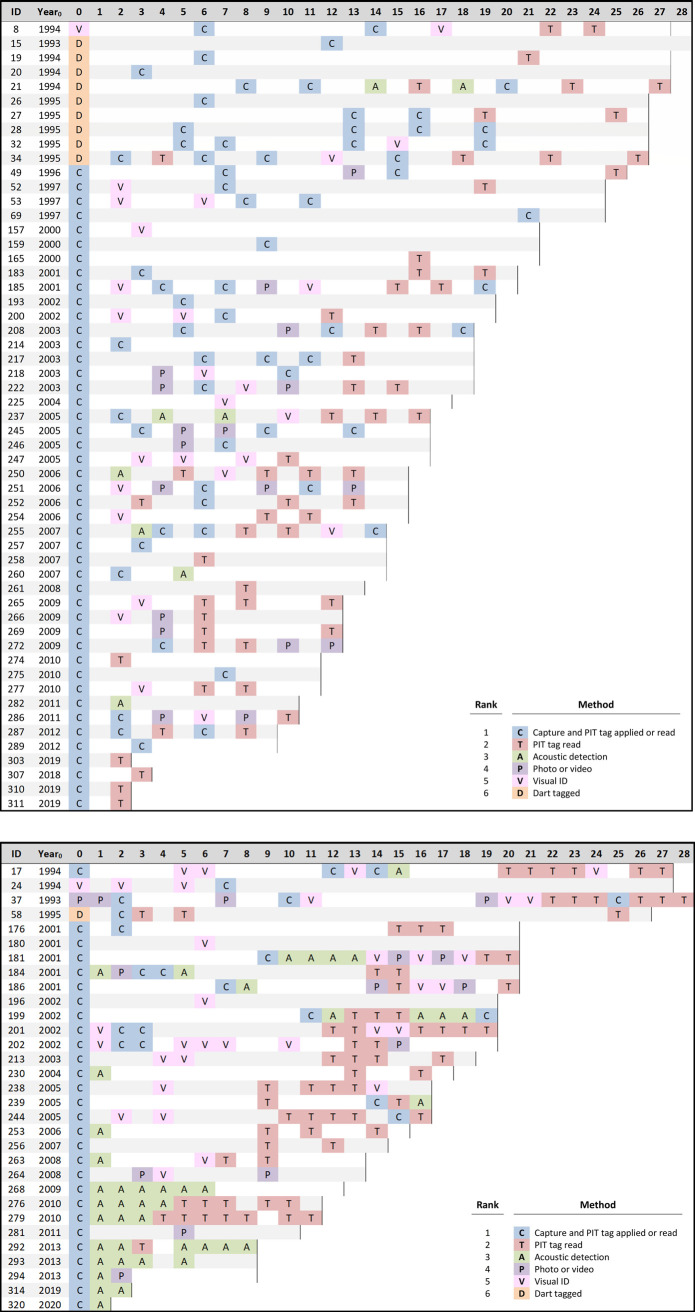
a. Resighting year and method for all returning female nurse sharks, 1993–2021 (N = 55). For shark-years in which multiple resighting methods are available, we show the most verifiable method as ranked in the legend. Vertical bars indicate the last year that a shark could have been resighted (through 2021). Shark #69 was originally tagged as a 138 cm juvenile. b. Resighting year and method for all returning male nurse sharks, 1993–2021 (N = 31). For shark-years in which multiple resighting methods are available, we show the most verifiable method as ranked in the legend. Vertical bars indicate the last year sharks could have been resighted (through 2021). Shark #58 was originally tagged as a 104 cm juvenile.

**Fig 4:**
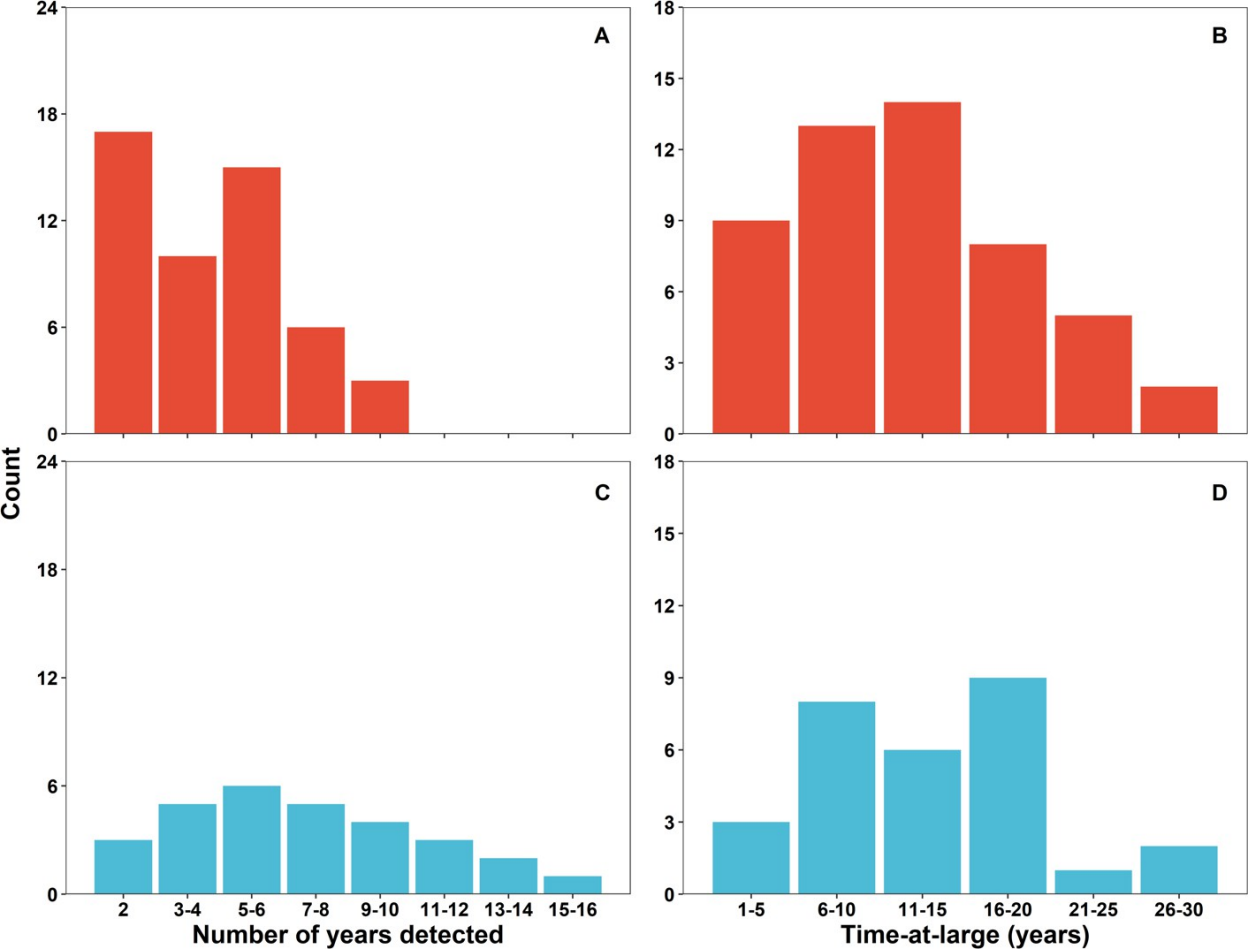
Nurse shark long-term use of the Dry Tortugas courtship and mating ground. For returning sharks only, a) and b) the number of mating seasons detected per shark over the course of the study and c) and d) the time-at-large (in years) for these sharks, defined as the most recent year of detection minus the first year in the study. Female = red fill (N = 51) and male = blue fill (N = 29). These returns reflect sharks tagged between 1993 and 2014, with return observations made through the mating season, 2021.

Of 118 sharks tagged from 1993 to 2014, at least 80 (68%) returned to the DTCMG in subsequent years during the June-July mating season. Nearly all returning animals did so in multiple years, and returns were detected using a combination of techniques (Fig 3A and 3B) described in the methods. The number of years detected was significantly different between sexes (Mann-Whitney test; U = 423.0, *p-value* = 0.001), with females and males having been detected for a median of 4 and 7 years, respectively. Returning females have been recorded on site in up to 10 separate mating seasons (Fig 4A), and multiple males have been recorded during 10 or more mating seasons, with a maximum of 16 mating seasons so far (Fig 4B). When considering the time from first identification to most recent detection, 59% (N = 47) of returning animals have been monitored for over 10 years, and 13% (N = 10) have been monitored for over 20 years (Fig 4C and 4D), with no significant difference between sexes (Mann-Whitney test; U = 645.5, *p-value* = 0.349).

The longest-monitored female sharks were recorded in 9 and 10 mating seasons over a span of 27 and 26 years (IDs #21 and #34, respectively; Fig 3A). One other female (ID #69) was measured as a juvenile in 1997 at 138 cm TL and recaptured 21 years later as a mating adult at 262 cm TL. The longest-monitored male sharks have been recorded in 16 and 14 mating seasons over 28 and 27 year spans (IDs #37 and #17, respectively; Fig 3B). Another male shark (ID #181) has returned every mating season for the last 12 consecutive years (Fig 3B). Of all returning sharks, at least 50% (40 of 80) have been detected in the last five years (2017–2021), indicating ongoing use of the DTCMG, and 34% (27 of 80) have been detected in the last three years (2019–2021). Of returning sharks tagged at least 20 years ago, 39% (11 of 28) have been detected in the last two years (2020–2021; Fig 3A and 3B).

### Reproductive cycles

**Fig 5:**
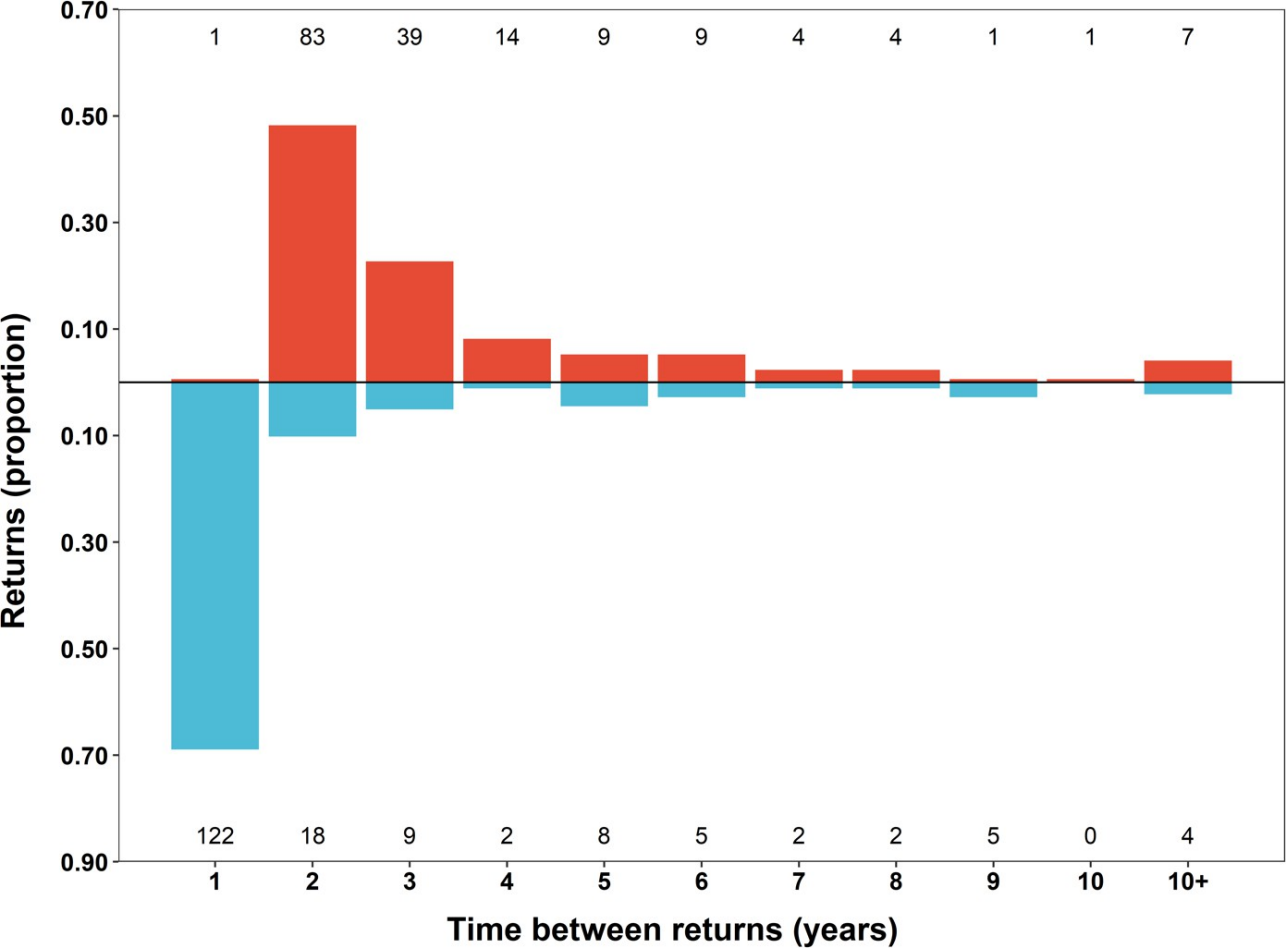
Annual return patterns of nurse sharks to the Dry Tortugas courtship and mating ground (DTCMG). The time between returns (in years) of nurse sharks returning to the DTCMG during the mating season (June-July). The proportion of total returns for females (red fill; N = 172 returns) and males (blue fill; N = 177 returns) are shown above and below the horizontal line at zero, respectively. Each year’s total number of individual returns is provided at the top and bottom of the panel for each sex, respectively. These returns reflect sharks tagged between 1993 and 2014, with return observations made through the mating season, 2021.

The periodicity of return events was significantly different between the sexes (Anderson-Darling test; *p-value* < 0.001), and the proportion of returns varied significantly across periods for females and males (Bayesian GLMM; χ_2_ = 348.8 and 391.8 and both *p-values* < 0.001, respectively). Return events after absences of five years or longer have been documented for both sexes. However, post-hoc multiple comparison tests revealed that the annual interval with the highest proportion of returns that were significantly different from all other periods was the two year interval in females (95% CI: 0.39–0.54) and the one year interval in males (95% CI: 0.56–0.70; Fig 5 and Table 1 in S2 File). Females also showed a significantly high proportion of returns after three years (95% CI: 0.16–0.28; Fig 5 and Table 1 in S2 File). After mating, females were nearly always absent from the mating ground in the subsequent mating season, whereas males usually returned annually (Fig 5).

For females, if we exclude return intervals of four years or longer, which may include a mating year that we missed, there were 83 return events after two years and 39 return events after three years, indicating that females mate on a triennial cycle in approximately 32% (39 of 122) of cases (Fig 5). Of 50 returning females that have carried tags for ten years or longer, 34% (17 of 50) have switched between 2-year and 3-year cycles at least once, and 18% (9 of 50) have switched cycles multiple times (Fig 3A). There was no apparent pattern to cycle switching and no sign that reproduction becomes more or less frequent as females age.

### Within-year use of the courtship and mating ground

**Fig 6:**
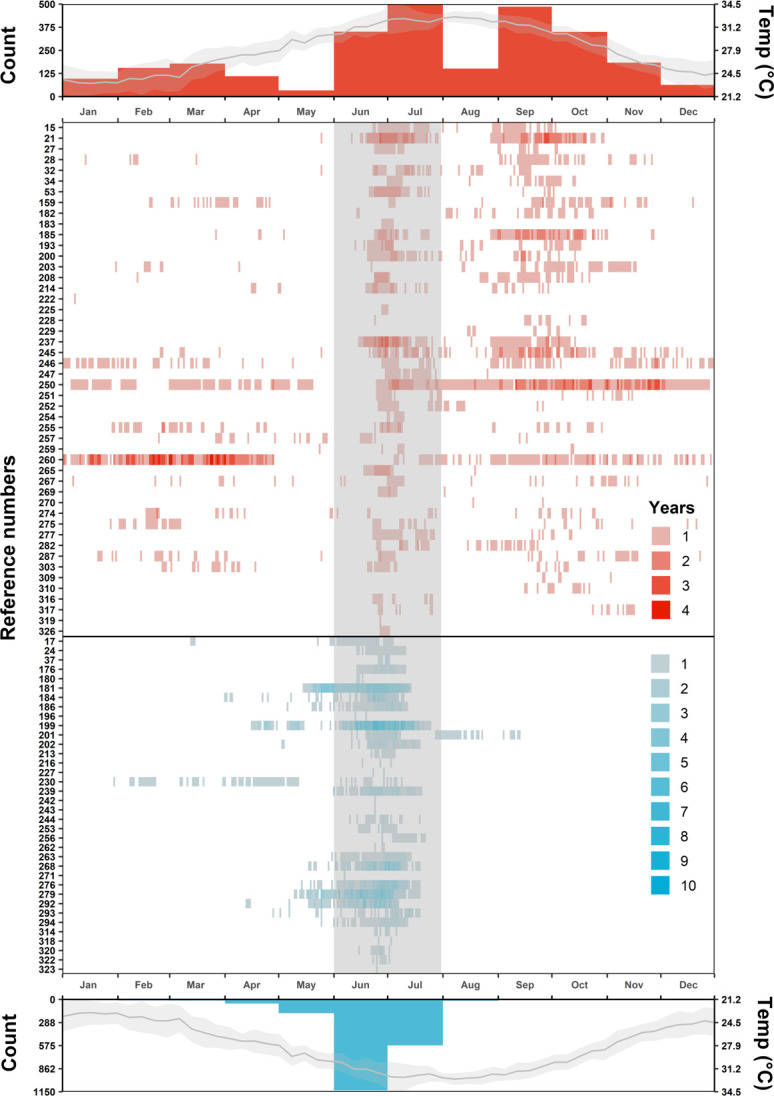
Monthly shark detections and water temperature on the Dry Tortugas courtship and mating ground. Acoustic detections (binned by day) with female and male sharks shown in red and blue, respectively. Detections (middle panels) are shaded darker for each year after tagging to indicate return year detections. The top and bottom histogram panels show the cumulative monthly number of daily detections across individuals for each sex. June and July are also highlighted (grey shading) in the middle panels to show when mating season occurs. Temperature lines show the mean (shading = standard deviation) maximum water temperature each week.

Acoustic detections show that females use the DTCMG in all months of the year, with peaks in the June-July mating season and September-October (Fig 6). August showed a relative drop in female detection days as mean seawater temperatures peaked at over 30°C (Fig 6). The month of May had the fewest number of days with detections, followed by December and January. Males used the DTCMG almost exclusively during June and July, with several males detected in May and some in April (Fig 6). Most male nurse sharks were never detected on the DTCMG from August through March (Fig 6).

**Fig 7:**
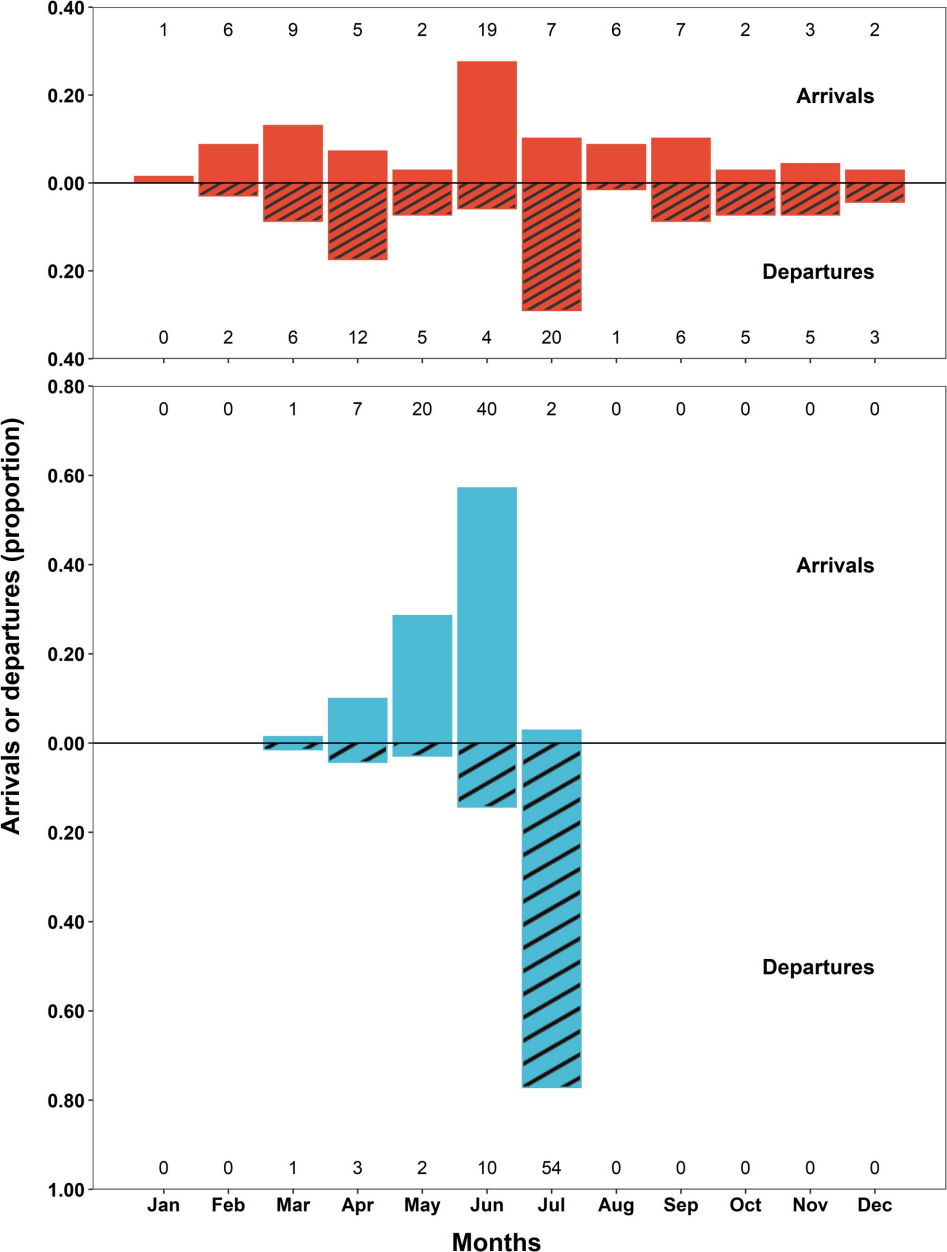
Seasonal arrivals and departures of nurse sharks on the Dry Tortugas courtship and mating ground. The proportions of shark arrivals (solid fill) and departures (fill with diagonal lines) are shown above and below the x-axis at zero, for females (red) and males (blue) respectively. Arrivals were defined as the first detection after a gap of at least 30 days without a detection. Departures were defined as the final detection before a gap of at least 30 days. The number of individual arrivals or departures for each sex in each month are also provided at the top and bottom of each panel, respectively. Arrivals and departures were omitted after the initial tagging event until the following April to remove any bias associated with tagging.

Arrival and departure patterns were significantly different between sexes throughout the year (Anderson-Darling test; *p-values* = 0.017 and 0.024, respectively). The proportion of arrivals and departures also varied significantly across months for both males (Bayesian GLMM; χ_2_ = 132.3 and 169.3 and both *p-values* < 0.001, respectively) and females (Bayesian GLMM; χ_2_ = 38.4 and 45.9 and both *p-values* < 0.001). Post-hoc multiple comparison tests revealed that female monthly arrival and departure proportions often overlapped in significance and that no single month had a significantly higher proportion than all others (Fig 7 and Tables 2 and 3 in S2 File). However, arrival and departure proportions were predominately highest in June (95% CI: 0.18–0.39) and July (95% CI: 0.19–0.40; Fig 7 and Tables 2 and 3 in S2 File) for females, respectively. Post-hoc multiple comparison tests for males identified the proportions of June arrivals and July departures as significantly higher than all other months (95% CIs: 0.45–0.68 and 0.66–0.86, respectively; Fig 7 and Tables 2 and 3 in S2 File).

During the mating season, most female arrivals took place in the last two weeks of June, with the earliest on June 12^th^ and the latest on July 8^th^, whereas male arrivals took place from the last week of May until the last week of June. Departures for both females and males peaked in the first week of July. Nearly all females had departed by the second week of July and nearly all males had departed by the third week.

### Within-season use of the courtship and mating ground

**Fig 8:**
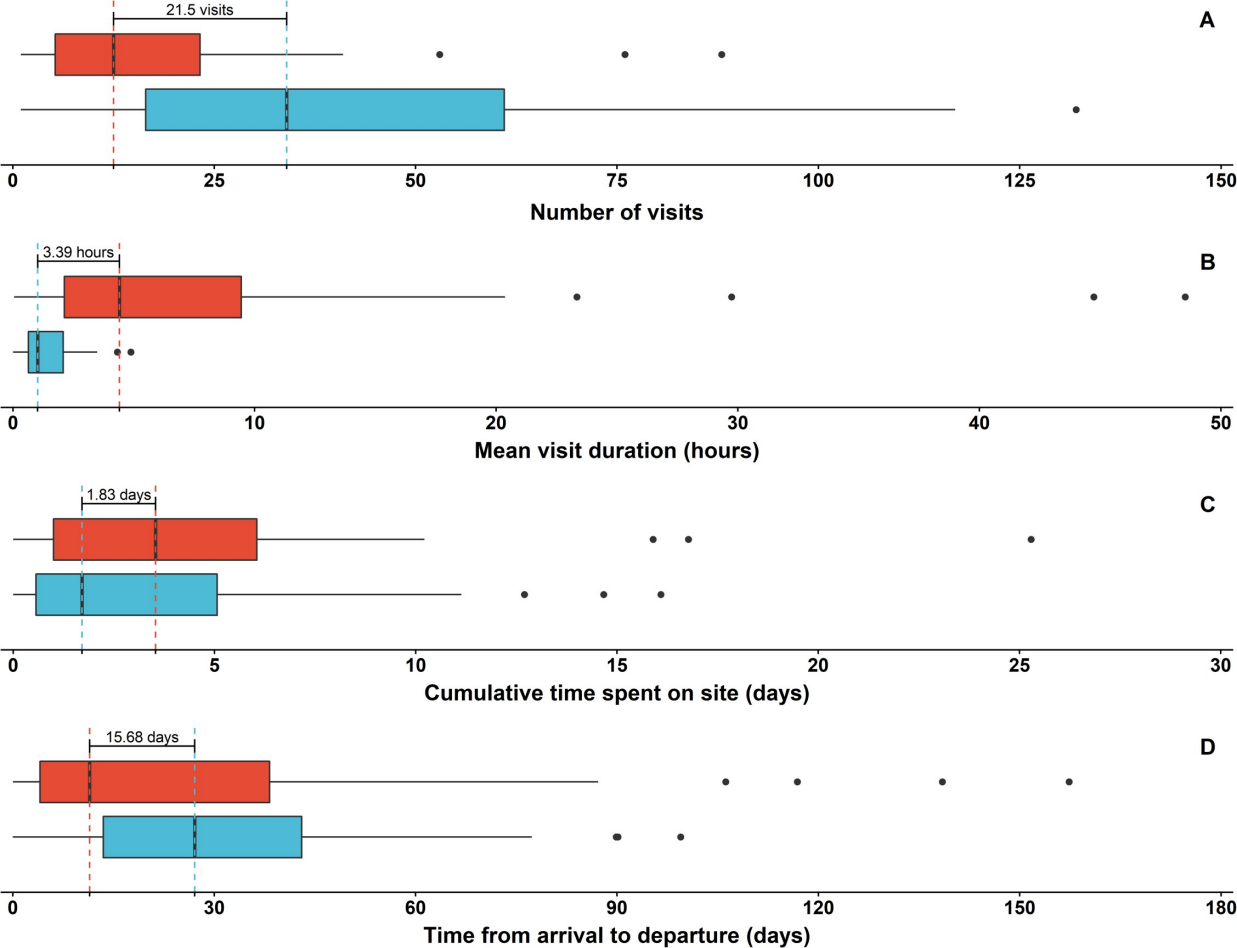
Nurse shark residence in the Dry Tortugas courtship and mating ground during mating season (June-July). Data based on acoustic detections of females (red) and males (blue), respectively. a) the mean number of visits to the site during mating seasons; b) mean visit duration (hours) for sharks during the mating season; c) the cumulative time (days) individual sharks were detected on site during mating seasons; and d) time from arrival to departure from the site during the mating season. A visit was defined as a sequence of acoustic detections without a gap of 60 minutes or more. Dashed lines indicate the median value for each metric, by sex, with the difference between the medians denoted with text.

Acoustic detections also revealed differences between the sexes in how the DTCMG site was used during the June-July mating season. Notably, there were significant differences between sexes in the number of visits and visit duration (GLMM; χ_2_ = 14.0 and 9.7 and *p-values* = < 0.001 and 0.002, respectively). Males tended to make more frequent visits of shorter duration (median = 34 visits of 1 h on average), whereas females made fewer visits but remained on site for longer periods per visit (median = 12.5 visits of 4.4 h on average; Fig 8). The average male visit duration during the mating season never exceeded 5 hours for any individual, whereas females often averaged 5–10 hour visits and even up to two full days without an absence longer than 60 minutes (Fig 8B). Though cumulative time spent on site was not significantly different between sexes (GLMM; χ_2_ = 1.9 and *p-value* = 0.164), the median female spent a cumulative total of 3.54 days on site during the mating season while the median male spent a cumulative total of 1.71 days on site (Fig 8C). The time between arrival and departure was also not significantly different between sexes (GLMM; χ_2_ = 0.1 and *p-value* = 0.730). But during the mating season, females stayed on site for a median of 11.43 days before departing, whereas males stayed on site for a median of 27.14 days between arrival and departure (Fig 8D).

**Fig 9:**
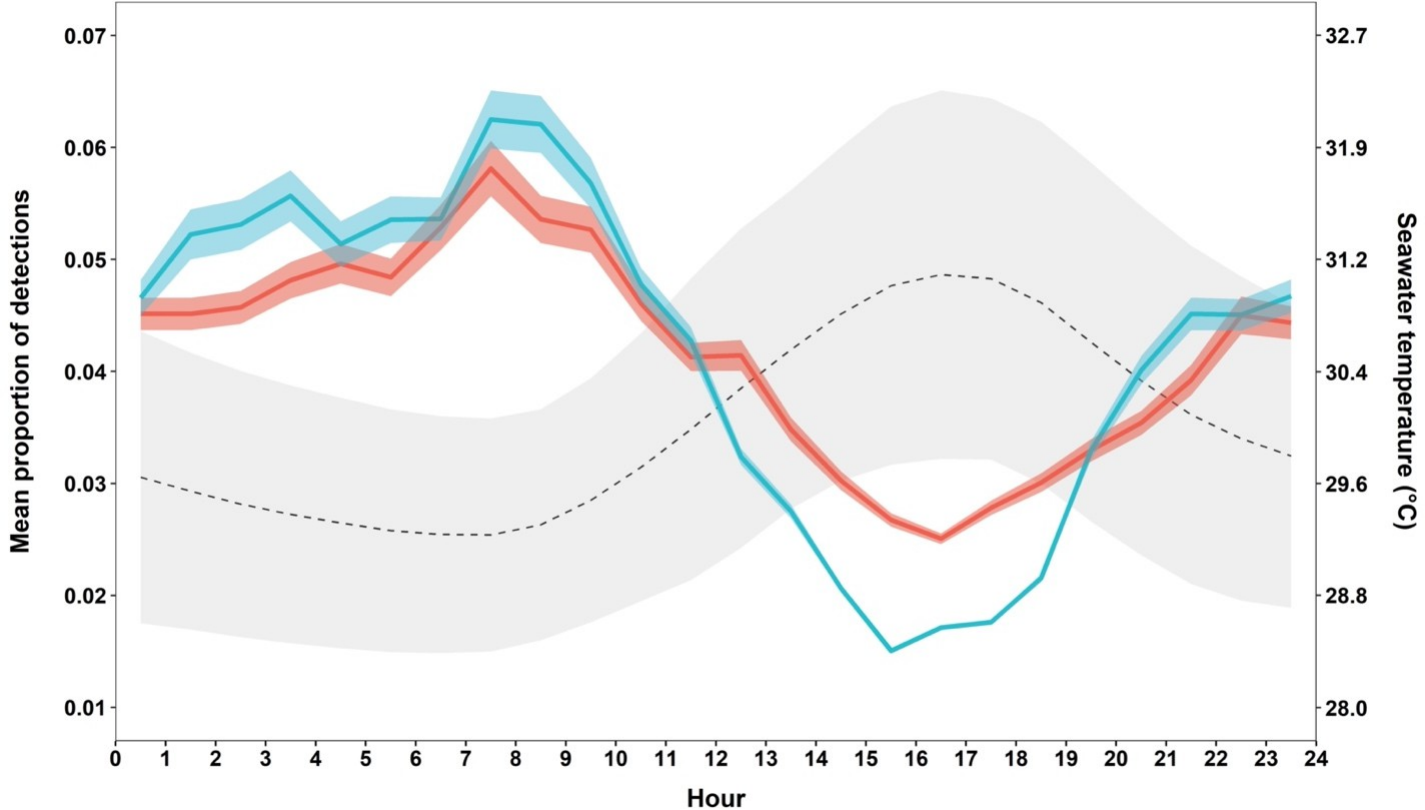
Nurse shark site use by time of day on the Dry Tortugas courtship and mating ground. The hourly proportion of nurse shark detections and hourly seawater temperature on site during the mating season (June–July). Detection proportions are shown as the mean (solid lines) and standard deviation (shaded areas) of hourly detection proportions across all years in this study for each sex (female = red; male = blue). Seawater temperature (°C) is shown as the hourly mean (black dashed line) and standard deviation (grey shaded area) of on-site temperatures during this study.

Both sexes visited the site at all hours of the day and night. Acoustic detections were variable across all hours of the day, with no strong diel pattern other than a tendency for detections to decrease gradually throughout the day as the water temperature in the DTCMG rose above 29.6°C (Fig 9). The lowest proportion of detections was from 15:00–17:00 for both sexes, after which the proportion of detections gradually increased from 17:00 to midnight and remained consistent throughout the night (Fig 9).

## Discussion

### Mating site fidelity

Our findings of long-term mating site fidelity show that the DTCMG is crucial for a subset of the nurse shark population in this region. To our knowledge, the return of individual sharks to the same mating site over multiple decades has not been previously documented, though it undoubtedly happens. Previous observations indicate that this site has been used by mating nurse sharks for over a century [33, 34] and probably much longer. Since recaptures are dependent on tag retention and detection, the number of returns we document here is likely an underestimate. These results emphasize not only the importance of this site but also the need to understand other essential habitats, known and unknown, utilized by elasmobranchs for feeding, mating, gestation, or nurseries [10, 17, 21, 29, 60]. Some of these long-lived animals are known to use the same areas repeatedly over many years, and our findings provide additional detail and duration to the extent of nurse shark mating site fidelity. This highlights the necessity of protecting critical habitat for elasmobranchs in addition to traditional fisheries regulations [21].

Although natal philopatry in this population is possible, it is difficult to demonstrate that females are returning to pup in their natal birth site without extensive study of both juveniles and adults [17]. Feldheim et al. [60] showed natal philopatry in lemon sharks (*Negaprion brevirostris*) through genetic profiling of 20 consecutive annual cohorts of neonates indicating that females return to give birth in Bimini, Bahamas, over multiple years and showing that at least six females born in Bimini returned to give birth there 14–17 years later. Using genetic parentage analysis based on microsatellite DNA, Mourier and Planes [61] found that blacktip reef sharks (*Carcharhinus melanopterus*) occasionally made long migrations over open ocean to give birth, with genetic siblings birthing in the same distant locations, providing strong circumstantial evidence for natal philopatry. Clark [62] used molecular techniques to show that Port Jackson shark females return to their natal birth site whereas males stray from their original birth site as juveniles but show strong philopatry to a breeding site as adults. After mating in June, many female nurse sharks were detected to reside locally in and around the DTCMG [43] through the fall season of parturition (October through December [45]), suggesting that they may give birth in the area. The authors have occasionally found neonates in and around the DTCMG, but there is no evidence that this is a nursery area or that nurse sharks utilize nurseries, as defined by Heupel et al. [10]. Given the punctuated nature of ovulation and the potential for a litter of pups to be birthed over a period of several weeks [39, 45], it is possible that neonates are distributed over a broad geographic area and do not aggregate. Although one female (ID #69) was tagged as a juvenile in 1997 and recaptured while mating in 2018 (Fig 3A), we cannot say with certainty where this shark was born or where it gave birth. More research sampling juveniles and using molecular techniques is needed to test whether natal philopatry exists in this population.

### Why this site: Geography and habitat

Carrier and Pratt [40] have described the habitat surrounding the mating ground in detail and the need for its seasonal closure [41]. One question yet to be answered is why this site, in particular, is so important for nurse shark reproduction. The Dry Tortugas contain a myriad of sand flats and low-lying islands [63] that would appear to provide similar habitat for sharks to refuge and mate in shallow water. Despite years of searching by the authors, monitoring via acoustic telemetry, and frequent surveys by researchers studying corals, fishes, birds, and sea turtles, no other nurse shark aggregation hot spots have been noted in the Dry Tortugas. The DTCMG may be unique to other sites in the amount of shelter and elevated water temperatures it provides in close proximity to deep water, but this requires further investigation.

Our lack of an unbiased sampling regime has, so far, prevented us from directly estimating the size of the population using this site. Considering that we typically detect an average of 26 sharks on site per mating season, and we estimate that approximately half of the sharks we encounter are tagged, this would produce a rough estimate of just over 50 sharks that use this site for reproduction every year. This is likely a very small fraction of the mating nurse sharks found within the known range of our tagged animals, which includes the waters of the Florida Keys, the western Bahamas, and the Florida peninsula north to at least South Carolina [43 and authors’ unpublished data]. Most nurse sharks in this range clearly do not use the DTCMG for mating and reproduction, but it is possible that they use other, more cryptic sites to which they show an equal level of site fidelity. For instance, nurse shark mating has been regularly observed at Cape Eleuthera, Bahamas [64] and Fernando de Noronha, Brazil [65]. Potential group courtship behavior has also been video recorded in the Marquesas Island group, Florida, approximately 75 km east of the DTCMG [43]. However, only one of our acoustically tagged sharks from the DTCMG was detected in three years of additional acoustic monitoring at these islands [authors’ unpublished data]. Most of these extant known breeding grounds are in extremely remote areas, and our work has shown how shark courtship and mating can be disturbed by the presence of vessels and humans [41]. It thus seems plausible that such breeding sites were more common before coastal development by humans.

### Why this site: Behavioral clues and the effect of depth and temperature

Carrier et al. [36] noted that female nurse sharks often attempted to avoid male mating advances on the DTCMG by arching their bodies away from the males and shoaling in the shallows and that copulation only occurred in water deeper than 0.5 m. Pratt and Carrier [24] reported that only 8% of observed mating events resulted in copulation and hypothesized that females use the shallows to enhance their ability to avoid certain males and select the mate of their choice. Although female mate selection may be enhanced in shallow water, comparative observations from deeper water are needed to confirm this hypothesis, and other factors may also be driving shallow water aggregation behavior.

Our finding of an autumn peak in the presence of post-mating females on the DTCMG (Fig 6), coinciding with a total absence of males, supports the hypothesis that females are using this site for behavioral thermoregulation during gestation. The nurse shark gestation period is known to last from June to November [45], and several elasmobranch species have been shown to seek warmer waters during gestation in an apparent effort to speed embryological development [e.g., 12, 13, 30, 66]. If this is the case, then thermoregulation may be the proximate driver of females using the DTCMG in June and July, seeking the shallows after they have copulated at least once in deeper water. Nurse shark females appear to ovulate and encapsulate ova sequentially over 2 to 3 weeks [45] and are known to have multiple paternity within broods [67, 68], so repeated mating events after the first insemination would be expected.

Neither mate selection nor behavioral thermoregulation explains the paucity of female detections on the site in late July and August, and it is possible that water temperatures over 30°C during this time exceed thermal tolerances, even for gestating females. This temperature-driven scenario is further supported by the fact that water temperatures in August are similar to those measured on the site during June-July afternoons when both sexes appear less likely to be detected in the shallows of the DTCMG.

Although males often arrive on the DTCMG earlier than females and are detected over a wider range of days, they do not appear to form any sort of aggregation that females visit (aka “a lek”) as has been suggested for white sharks [18]. On the contrary, females spend the most cumulative time on site while males make repeated, comparatively brief visits. Our direct observations confirm that females visit the site for longer periods and suggest that males typically visit for only a few minutes before moving on to other areas [24]. However, acoustic detections show mean visit durations of over three hours for males, indicating that they spend more time on site than we expected. During periods of perceived absence they may often remain nearby, staying in 2 to 3 m of water within the range of acoustic receivers but out of sight for human observers.

### Reproductive cycles

The lack of consecutive year returns for females provides strong evidence that they do not reproduce annually and also indicates that their presence in the DTCMG during mating season is a valid proxy for fertilization and pregnancy. The different pattern of annual returns for males and females in this population was initially reported by Pratt and Carrier [24] and is consistent with anatomical evidence indicating a biennial reproductive cycle for females and annual reproduction for males [45]. However, our long-term study of live animals has revealed that females mate on a triennial cycle in approximately 32% of cases and that individual females may switch cycles more than once. Based on methods described by Driggers et al. [69], we used a potential reproductive lifetime output (PRLO) analysis to show that the pattern we observed would reduce the total number of pups produced by a female from 612 to 544, a reduction of 11.1% compared to a strict biennial cycle (see Table 1 in S1 File for details).

Although variability in female reproductive cycles has been noted in other elasmobranchs, it has consisted of the simultaneous occurrence of annual and biennial reproduction in different females [70, 71]. Our study appears to be the first to show individuals of a biennial species occasionally reproducing triennially and the first to document individual females switching reproductive cycles. This phenomenon would be extremely difficult to detect in traditional anatomical studies of elasmobranch reproduction but, if common, would reduce the overall fecundity of biennial species by a percentage that increases with brood size and number of female lifetime reproductive events.

### Life span

Our findings nearly double the previously published maximum life span for nurse sharks from 25 years [72] to well over 40 years. Although age and growth data are lacking for this species, if we start with the largest juvenile size classes for which data are available (~144 cm at an estimated age of 5 years by Carrier and Luer [73] and assume a growth rate of 5–10 cm per year until size at maturity (214 cm for males; 223–231 cm for females [45]), age at maturity would range from the mid-teens to the mid-twenties for nurse sharks. If we conservatively assume an age at maturity of 15 years, then the longest monitored female in our study would be 43 years old. If these sharks mature in their twenties, then several of our study animals are likely to be in their late 40s to over 50 years old. The fact that 39% (11/28) of returning sharks that were originally tagged > 20 years ago (most as adults) have been detected on site in the last two years indicates that many of our oldest known sharks are still alive and reproductively active, and we expect our maximum longevity estimate to increase as we continue monitoring. Unpublished data show that there are now several nurse sharks in aquariums that are in their 30s and 40s, with the oldest being 43 years old [J. Janssen, National Aquarium, personal communication].

### Conservation

Elasmobranchs are susceptible to overfishing and population loss due to their conservative life history traits and are in decline in the Caribbean [74] and worldwide [4, 75]. There is a pressing need to incorporate knowledge of mating systems in population assessments and field research on spatial and temporal scales of reproduction [6]. Although nurse sharks are not targeted by fisheries in this region, they are considered a nuisance by many fishermen and are sometimes killed at sea [45]. They are considered to be at risk of extinction in Brazil [76] and have recently been assessed as “vulnerable” in the Atlantic, where they are likely to have undergone a population reduction of 30–49% over the past 90 years due to fishing pressure, habitat loss, and the impacts of climate change [77].

Continued monitoring of the DTCMG will allow us to see the impacts of climate change on this population as coral reefs decline and the sea level continues to rise. Storms and shifting sands have dramatically reshaped the islands and study site, and coral heads that were once key locations for mating or juvenile refuges are now buried. Ongoing studies are exploring the energetics of courtship and mating, patterns in mate selection and attraction, and thermoregulatory behavior of females in reproductive versus resting years. The repeated, long-term use of the DTCMG by adult nurse sharks and the clear, shallow environment make this site a natural laboratory providing a unique opportunity to address questions about shark biology that are difficult or impossible to answer from traditional anatomical studies.

## Supporting information

S1 File Potential reproductive lifetime output (PRLO). This file includes a description and details of PRLO for a hypothetical female nurse shark and an annotated example of the calculations used to estimate the output.
(DOCX)

S2 File Post-hoc multiple comparisons. This file includes the results of post-hoc multiple comparisons from generalized linear mixed models (in a Bayesian setting) used to evaluate patterns in the annual returns of nurse sharks and arrival and departure throughout the year at the Dry Tortugas Courtship and Mating Grounds.
(DOCX)
