# Supplementary material for: Long-term use of a shark breeding ground: Three decades of mating site fidelity in the nurse shark, Ginglymostoma cirratum
Source: PLoS One. 2022 Oct 17;17(10):e0275323. doi: 10.1371/journal.pone.0275323 (PMC9576040; doi:10.1371/journal.pone.0275323)
Supplement: S1 File — This file includes a description and details of PRLO for a hypothetical female nurse shark and an annotated example of the calculations used to estimate the output. (DOCX) [file pone.0275323.s001.docx]

**S1 File. Potential reproductive lifetime output (PRLO).** This file includes a description and details of PRLO for a hypothetical female nurse shark and an annotated example of the calculations used to estimate the output.

To explore the difference in potential reproductive lifetime output (PRLO) of a nurse shark exhibiting two reproductive cycle scenarios, we calculated the hypothetical total number of offspring attributable to an individual shark under both scenarios following Driggers et al. (2020; see for more details). PRLO was calculated using the following nurse shark life-history characteristics: age at maturity and maximum lifespan of 15 and 50-years, respectively (this study), a brood size of 34 pups, and an approximately six-month gestation period (Castro 2000). We considered two reproductive scenarios, including a biennial-only cycle (Castro 2000) and a combination of biennial and triennial cycles that we estimate to occur in 68% and 32% of reproductive events, respectively. Similar to Driggers et al. (2020), we assumed nil mortality of the female and her offspring throughout her lifetime and did not consider the relationship between age or length and brood size. We also did not account for any potential brood size disparities resulting from energetic differences between the two reproductive cycles.

Castro, J. I. 2000. The biology of the nurse shark, *Ginglymostoma cirratum*, off the Florida east

coast and the Bahama Islands. Environmental Biology of Fishes, 58(1), 1-22.

Driggers III, W. B., Hoffmayer, E. R., Campbell, M. D., Jones, C. M., Hannan, K. M., and

Sulikowski, J. A. 2020. Spatial variability in the fecundity of Atlantic sharpnose sharks

(*Rhizoprionodon terraenovae*) in the northern Gulf of Mexico. Fishery Bulletin, 118(1),

51-63.

**Table 1.** **Potential reproductive lifetime output (PRLO) of a hypothetical female nurse shark (*Ginglymostoma cirratum*)**. This scenario assumes the nurse shark follows a biennial-only reproductive cycle or a combination of biennial and triennial cycles (68 and 32% of gestations, respectively). The columns on either side of the brood column represent the number of offspring attributable to the original female nurse shark (first generation) and her female offspring (second and third generations) throughout her lifetime. Next to each brood size is also the age of the original nurse shark (in years; shown in subscript) associated with the respective generation and brood. The total number of offspring produced by the original nurse shark (dashed lines) is provided in grey-highlighted boxes. Total PRLO is the cumulative sum of offspring under each reproductive scenario attributable to the original nurse shark during her lifetime. The life-history characteristics and assumptions used in PRLO calculations are provided at the bottom of the table.

| **Biennial-only**  **reproductive cycle** | | | |  |  | |  | **Combination of biennial and triennial**  **reproductive cycles** | | |
| --- | --- | --- | --- | --- | --- | --- | --- | --- | --- | --- |
| **Generation 3** | **Generation 2** | | **Generation 1** |  | **Brood** | |  | **Generation 1** | **Generation 2** | **Generation 3** |
|  |  | |  |  |  | |  |  |  |  |
| 9,826_46.0_ | 578_30.5_ | | 34_15.0_ |  | 1 | |  | 34_15.0_ | 578_30.5_ | 9,826_46.0_ |
| 9,826_48.0_ | 578_32.5_ | | 34_17.0_ |  | 2 | |  | 34_17.3_ | 578_32.8_ | 9,826_48.3_ |
| 9,826_50.0_ | 578_34.5_ | | 34_19.0_ |  | 3 | |  | 34_19.6_ | 578_35.1_ |  |
|  | 578_36.5_ | | 34_21.0_ |  | 4 | |  | 34_22.0_ | 578_37.5_ |  |
|  | 578_38.5_ | | 34_23.0_ |  | 5 | |  | 34_24.3_ | 578_39.8_ |  |
|  | 578_40.5_ | | 34_25.0_ |  | 6 | |  | 34_26.6_ | 578_42.1_ |  |
|  | 578_42.5_ | | 34_27.0_ |  | 7 | |  | 34_28.9_ | 578_44.4_ |  |
|  | 578_44.5_ | | 34_29.0_ |  | 8 | |  | 34_31.2_ | 578_46.7_ |  |
|  | 578_46.5_ | | 34_31.0_ |  | 9 | |  | 34_33.6_ | 578_49.1_ |  |
|  | 578_48.5_ | | 34_33.0_ |  | 10 | |  | 34_35.9_ |  |  |
|  |  | | 34_35.0_ |  | 11 | |  | 34_38.2_ |  |  |
|  |  | | 34_37.0_ |  | 12 | |  | 34_40.5_ |  |  |
|  |  | | 34_39.0_ |  | 13 | |  | 34_42.8_ |  |  |
|  |  | | 34_41.0_ |  | 14 | |  | 34_45.2_ |  |  |
|  |  | | 34_43.0_ |  | 15 | |  | 34_47.5_ |  |  |
|  |  | | 34_45.0_ |  | 16 | |  | 34_49.8_ |  |  |
|  |  | | 34_47.0_ |  | 17 | |  | **544** |  |  |
|  |  | | 34_49.0_ |  | 18 | |  |  |  |  |
|  |  | | **612** |  |  | |  |  |  |  |
|  |  | |  |  |  | |  |  |  |  |
| **Total PRLO = 35,870** | | | |  |  | |  | **Total PRLO = 25,398** | | |
|  | | | | | | | | | | |
| **Nurse shark life-history characteristics** | | | | | | | | | | |
| Age at maturity | | 15 years (this study) | | | | | | | | |
| Maximum age | | 50 years (this study) | | | | | | | | |
| Brood size | | 34 pups (Castro 2000) | | | | | | | | |
| Gestation period | | 0.5 years (Castro 2000) | | | | | | | | |
| Reproductive cycle | | Biennial-only (Castro 2000) | | | | | | | | |
|  | | Biennial (68% of reproductive events; this study) | | | | | | | | |
|  | | Triennial (32% of reproductive events; this study) | | | | | | | | |
|  | | | | | |  | | | | |
| **Additional assumptions** | | | | | |  | | | | |
| Nil mortality in female offspring throughout the original female’s lifetime | | | | | | | | | | |
| No relationship between brood size and animal length or age | | | | | | | | | | |
| No relationship between brood size and reproductive scenario (biennial-only vs. combination of cycles) | | | | | | | | | | |

Below are annotated examples of calculations from Table 1. Examples reflect the PRLO of a biennial-only reproductive cycle female, but the same calculations apply to a female displaying a combination of biennial and triennial cycles (i.e., right-hand side of the table).

| **General Notation** | |
| --- | --- |
| $\boldsymbol{B}_{\boldsymbol{A}^{\boldsymbol{G}\boldsymbol{1}}}$ | |
| $B=$ | $brood size (in number offspring)$ |
| $A^{G1}=$ | $age of the original female associated with each generation and brood$ |

**Generation 1**

This column represents the offspring produced by the original female – the 1^st^ generation – throughout her lifetime.

| *E.g., Generation 1 – Brood 1 –* the first offspring produced by the original female. | |
| --- | --- |
| **34_15.0_** | |
| $B=$ | $brood size$ |
| $=$ | $34 offspring$ |
| $A^{G1}=$ | $age of maturity$ |
| $=$ | $15.0 years old$ |

| *E.g., Generation 1 – Broods 2 to 18 –* the additional broods produced by the original female. | |
| --- | --- |
| **34_17.0_ … 34_n_** | |
| $B=$ | $brood size$ |
| $=$ | $34 offspring$ |
| $A^{G1}=$ | $orginal female age from previous reproductive event+ reproductive cycle$ |
| $=$ | $15+2$ |
| $=$ | $17 years old$ |

**Generation 2**

This column represents the offspring produced by the female offspring of broods from the original female – the 2^nd^ generation – throughout the original female’s lifetime.

| *E.g., Generation 2 – Brood 1* **–** the offspring produced by the 1^st^ generation females related to the original female’s first brood. | |
| --- | --- |
| **578_30.5_** | |
| $B=$ | ${brood size of the original female}/{sex ratio \times}individual brood size$ |
| $=$ | ${34}/2\times34$ |
| $=$ | $578 offspring$ |
| $A^{G1}=$ | $orginal female^{'}s age at first brood+gestation period+age at maturity$ |
| $=$ | $15+0.5+ 15$ |
| $=$ | $30.5 years old$ |

| *E.g., Generation 2 – Broods 2 to 10 –* the offspring produced by the 1^st^ generation females in relation to the original female’s additional broods. | |
| --- | --- |
| **578_32.5_ … 578_n_** | |
| $B=$ | ${brood size of the original female}/{sex ratio \times}individual brood size$ |
| $=$ | ${34}/2\times34$ |
| $=$ | $578 offspring$ |
| $A^{G1}=$ | $orginal female^{'}s age per brood+gestation period+age at maturity$ |
| $=$ | $17+0.5+ 15$ |
| $=$ | $32.5 years old$ |

**Generation 3**

This column represents the offspring produced by the female offspring of broods from the 2^nd^ generation females – the 3^rd^ generation – throughout the original female’s lifetime.

| *E.g., Generation 3 – Brood 1* **–** the offspring produced by the 2^nd^ generation females related to the original female’s first brood. | |
| --- | --- |
| **9,826_46_** | |
| $B=$ | ${brood size of 2nd generation females}/{sex ratio \times}individual brood size$ |
| $=$ | ${578}/2\times34$ |
| $=$ | $9,826 offspring$ |
| $A^{G1}=$ | $original female age when 2nd generation females from her first brood become mature +$  $gestation period+age at maturity$ |
| $=$ | $30.5+0.5+ 15$ |
| $=$ | $46 years old$ |

| *E.g., Generation 3 – Broods 2 and 3 –* the offspring produced by 2^nd^ generation females in relation to the original female’s additional broods. | |
| --- | --- |
| **9,826_48_ … 9,826_n_** | |
| $B=$ | ${brood size of 2nd generation females}/{sex ratio \times}individual brood size$ |
| $=$ | ${578}/2\times34$ |
| $=$ | $9,826 offspring$ |
| $A^{G1}=$ | $original female age when 2nd generation females per brood become mature +$  $gestation period+age at maturity$ |
| $=$ | $32.5+0.5+ 15$ |
| $=$ | $48 years old$ |
