# Supplementary material for: Long-term use of a shark breeding ground: Three decades of mating site fidelity in the nurse shark, Ginglymostoma cirratum
Source: PLoS One. 2022 Oct 17;17(10):e0275323. doi: 10.1371/journal.pone.0275323 (PMC9576040; doi:10.1371/journal.pone.0275323)
Supplement: S2 File — This file includes the results of post-hoc multiple comparisons from generalized linear mixed models (in a Bayesian setting) used to evaluate patterns in the annual returns of nurse sharks and arrival and departure throughout the year at the Dry Tortugas Courtship and Mating Grounds. (DOCX) [file pone.0275323.s002.docx]

**S2 File.** **Post-hoc multiple comparisons.** This file includes the results of post-hoc multiple comparisons from generalized linear mixed models (in a Bayesian setting) used to evaluate patterns in the annual returns of nurse sharks and arrival and departure throughout the year at the Dry Tortugas Courtship and Mating Grounds.

**Table 1.** Multiple comparisons from generalized linear mixed models (in a Bayesian setting; see “Methods” section for more details) evaluating the proportion of nurse shark returns to the Dry Tortuga Courtship and Mating Grounds during the mating season as a function of the time between return years. Models were run separately for each sex, and the post-hoc multiple comparisons were performed with Tukey’s p-value adjustment method. Estimated marginal means, standard errors (± SE), and the 95% confidence intervals (CI) of return proportions are provided. Return year proportions significantly different from others based on an α-level of 0.05 are denoted with unique capital letters.

|  | **Female** | | |  | **Male** | | |
| --- | --- | --- | --- | --- | --- | --- | --- |
| **Return years** | **Groups** | **Probability ± SE** | **95% CI** |  | **Groups** | **Probability ± SE** | **95% CI** |
| 1 | C | 0.024 ± 0.005 | 0.015 - 0.037 |  | A | 0.628 ± 0.035 | 0.557 - 0.695 |
| 2 | A | 0.462 ± 0.038 | 0.389 - 0.536 |  | B | 0.103 ± 0.023 | 0.066 - 0.157 |
| 3 | B | 0.213 ± 0.031 | 0.159 - 0.279 |  | B | 0.053 ± 0.017 | 0.028 - 0.097 |
| 4 | C | 0.074 ± 0.019 | 0.044 - 0.122 |  | B | 0.014 ± 0.009 | 0.004 - 0.047 |
| 5 | C | 0.048 ± 0.015 | 0.025 - 0.089 |  | B | 0.047 ± 0.016 | 0.024 - 0.090 |
| 6 | C | 0.048 ± 0.015 | 0.025 - 0.089 |  | B | 0.031 ± 0.013 | 0.013 - 0.069 |
| 7 | C | 0.0230 ± 0.010 | 0.010 - 0.055 |  | B | 0.014 ± 0.009 | 0.004 - 0.047 |
| 8 | C | 0.0230 ± 0.010 | 0.010 - 0.055 |  | B | 0.014 ± 0.009 | 0.004 - 0.047 |
| 9 | C | 0.011 ± 0.006 | 0.003 - 0.034 |  | B | 0.033 ± 0.014 | 0.014 - 0.074 |
| 10 | C | 0.011 ± 0.006 | 0.003 - 0.034 |  | B | 0.004 ± 0.005 | 0.000 - 0.036 |
| 11 | C | 0.007 ± 0.005 | 0.002 - 0.028 |  | B | 0.010 ± 0.007 | 0.002 - 0.043 |
| 12 | C | 0.016 ± 0.008 | 0.005 - 0.044 |  | B | 0.011 ± 0.008 | 0.002 - 0.048 |
| 13 | C | 0.017 ± 0.009 | 0.006 - 0.048 |  | B | 0.011 ± 0.009 | 0.002 - 0.050 |
| 14 | C | 0.008 ± 0.005 | 0.002 - 0.030 |  | B | 0.005 ± 0.006 | 0.001 - 0.045 |
| 15 | C | 0.013 ± 0.008 | 0.004 - 0.042 |  | B | 0.005 ± 0.006 | 0.001 - 0.046 |
| 16 | C | 0.014 ± 0.009 | 0.004 - 0.047 |  | B | 0.005 ± 0.006 | 0.001 - 0.047 |
| 17 | C | 0.010 ± 0.007 | 0.002 - 0.040 |  | B | 0.006 ± 0.007 | 0.001 - 0.055 |
| 18 | C | 0.010 ± 0.007 | 0.002 - 0.040 |  | B | 0.006 ± 0.007 | 0.001 - 0.056 |
| 19 | C | 0.011 ± 0.008 | 0.002 - 0.047 |  | B | 0.006 ± 0.007 | 0.001 - 0.060 |
| 20 | C | 0.011 ± 0.009 | 0.003 - 0.049 |  | B | 0.022 ± 0.017 | 0.005 - 0.098 |
| 21 | C | 0.021 ± 0.014 | 0.005 - 0.076 |  | B | 0.015 ± 0.019 | 0.001 - 0.153 |

**Table 2.** Multiple comparisons from generalized linear mixed models (in a Bayesian setting; see “Methods” section for more details) evaluating the proportion of nurse shark arrivals to the Dry Tortuga Courtship and Mating Grounds as a function of the time throughout the year (in months). Models were run separately for each sex, and the post-hoc multiple comparisons were performed with Tukey’s p-value adjustment method. Estimated marginal means, standard errors (± SE), and the 95% confidence intervals (CI) of arrival proportions are provided. Monthly proportions significantly different from others based on an α-level of 0.05 are denoted with unique capital letters.

|  | **Female** | | |  | **Male** | | |
| --- | --- | --- | --- | --- | --- | --- | --- |
| **Months** | **Groups** | **Probability ± SE** | **95% CI** |  | **Groups** | **Probability ± SE** | **95% CI** |
| Jan | B | 0.034 ± 0.017 | 0.012 - 0.090 |  | C | 0.016 ± 0.007 | 0.007 - 0.037 |
| Feb | AB | 0.085 ± 0.033 | 0.039 - 0.177 |  | C | 0.009 ± 0.007 | 0.002 - 0.044 |
| Mar | AB | 0.128 ± 0.040 | 0.068 - 0.229 |  | C | 0.015 ± 0.013 | 0.003 - 0.076 |
| Apr | AB | 0.071 ± 0.031 | 0.030 - 0.160 |  | BC | 0.091 ± 0.034 | 0.044 - 0.182 |
| May | B | 0.029 ± 0.020 | 0.008 - 0.105 |  | B | 0.286 ± 0.054 | 0.192 - 0.402 |
| Jun | A | 0.272 ± 0.053 | 0.180 - 0.387 |  | A | 0.571 ± 0.059 | 0.454 - 0.682 |
| Jul | AB | 0.100 ± 0.036 | 0.048 - 0.195 |  | C | 0.026 ± 0.018 | 0.007 - 0.094 |
| Aug | AB | 0.085 ± 0.033 | 0.039 - 0.177 |  | C | 0.009 ± 0.007 | 0.002 - 0.044 |
| Sep | AB | 0.100 ± 0.036 | 0.048 - 0.195 |  | C | 0.009 ± 0.007 | 0.002 - 0.044 |
| Oct | B | 0.029 ± 0.020 | 0.008 - 0.105 |  | C | 0.009 ± 0.007 | 0.002 - 0.044 |
| Nov | B | 0.043 ± 0.024 | 0.014 - 0.123 |  | C | 0.009 ± 0.007 | 0.002 - 0.044 |
| Dec | B | 0.029 ± 0.020 | 0.008 - 0.105 |  | C | 0.009 ± 0.007 | 0.002 - 0.044 |

**Table 3.** Multiple comparisons from generalized linear mixed models (in a Bayesian setting; see “Methods” section for more details) evaluating the proportion of nurse shark departures from the Dry Tortuga Courtship and Mating Grounds as a function of the time throughout the year (in months). Models were run separately for each sex, and the post-hoc multiple comparisons were performed with Tukey’s p-value adjustment method. Estimated marginal means, standard errors (± SE), and the 95% confidence intervals (CI) of departure proportions are provided. Monthly proportions significantly different from others based on an α-level of 0.05 are denoted with unique capital letters.

|  | **Female** | | |  | **Male** | | |
| --- | --- | --- | --- | --- | --- | --- | --- |
| **Months** | **Groups** | **Probability ± SE** | **95% CI** |  | **Groups** | **Probability ± SE** | **95% CI** |
| Jan | B | 0.025 ± 0.014 | 0.008 - 0.074 |  | C | 0.015 ± 0.007 | 0.006 - 0.036 |
| Feb | B | 0.029 ± 0.020 | 0.007 - 0.105 |  | C | 0.008 ± 0.007 | 0.002 - 0.043 |
| Mar | AB | 0.085 ± 0.033 | 0.039 - 0.177 |  | BC | 0.015 ± 0.012 | 0.003 - 0.075 |
| Apr | AB | 0.171 ± 0.045 | 0.099 - 0.277 |  | BC | 0.038 ± 0.022 | 0.012 - 0.112 |
| May | AB | 0.071 ± 0.031 | 0.030 - 0.159 |  | BC | 0.026 ± 0.018 | 0.007 - 0.094 |
| Jun | B | 0.057 ± 0.027 | 0.021 - 0.141 |  | B | 0.143 ± 0.042 | 0.079 - 0.246 |
| Jul | A | 0.285 ± 0.054 | 0.192 - 0.402 |  | A | 0.771 ± 0.050 | 0.659 - 0.855 |
| Aug | B | 0.015 ± 0.014 | 0.002 - 0.088 |  | C | 0.008 ± 0.007 | 0.002 - 0.043 |
| Sep | AB | 0.085 ± 0.033 | 0.039 - 0.177 |  | C | 0.008 ± 0.007 | 0.002 - 0.043 |
| Oct | AB | 0.071 ± 0.031 | 0.030 - 0.159 |  | C | 0.008 ± 0.007 | 0.002 - 0.043 |
| Nov | AB | 0.071 ± 0.031 | 0.030 - 0.159 |  | C | 0.008 ± 0.007 | 0.002 - 0.043 |
| Dec | B | 0.043 ± 0.024 | 0.014 - 0.123 |  | C | 0.008 ± 0.007 | 0.002 - 0.043 |
